# Amphiphilic Polymeric Micelles Based on Deoxycholic Acid and Folic Acid Modified Chitosan for the Delivery of Paclitaxel

**DOI:** 10.3390/ijms19103132

**Published:** 2018-10-12

**Authors:** Liang Li, Na Liang, Danfeng Wang, Pengfei Yan, Yoshiaki Kawashima, Fude Cui, Shaoping Sun

**Affiliations:** 1 Key Laboratory of Chemical Engineering Process & Technology for High-efficiency Conversion, College of Heilongjiang Province, School of Chemistry and Material Science, Heilongjiang University, Harbin 150080, China; lliang1991001@163.com (L.L.); dfwang626@163.com (D.W.); yanpf@vip.sina.com (P.Y.); 2 Key Laboratory of Photochemical Biomaterials and Energy Storage Materials, Heilongjiang Province, College of Chemistry & Chemical Engineering, Harbin Normal University, Harbin 150025, China; liangna528@163.com; 3 Department of Pharmaceutical Engineering, School of Pharmacy, Aichi Gakuin University, Nagoya 464-8650, Japan; sykawa123@163.com; 4 School of Pharmacy, Shenyang Pharmaceutical University, Shenyang 110016, China; syphucuifude@163.com

**Keywords:** chitosan, deoxycholic acid, folic acid, amphiphilic polymer, micelles, paclitaxel

## Abstract

The present investigation aimed to develop a tumor-targeting drug delivery system for paclitaxel (PTX). The hydrophobic deoxycholic acid (DA) and active targeting ligand folic acid (FA) were used to modify water-soluble chitosan (CS). As an amphiphilic polymer, the conjugate FA-CS-DA was synthesized and characterized by Proton nuclear magnetic resonance (^1^H-NMR) and Fourier-transform infrared spectroscopy (FTIR) analysis. The degree of substitutions of DA and FA were calculated as 15.8% and 8.0%, respectively. In aqueous medium, the conjugate could self-assemble into micelles with the critical micelle concentration of 6.6 × 10^−3^ mg/mL. Under a transmission electron microscope (TEM), the PTX-loaded micelles exhibited a spherical shape. The particle size determined by dynamic light scattering was 126 nm, and the zeta potential was +19.3 mV. The drug loading efficiency and entrapment efficiency were 9.1% and 81.2%, respectively. X-Ray Diffraction (XRD) analysis showed that the PTX was encapsulated in the micelles in a molecular or amorphous state. In vitro and in vivo antitumor evaluations demonstrated the excellent antitumor activity of PTX-loaded micelles. It was suggested that FA-CS-DA was a safe and effective carrier for the intravenous delivery of paclitaxel.

## 1. Introduction

Paclitaxel (PTX) is an important clinical chemotherapeutic drug that exhibits strong antitumour activity against a variety of cancer types. However, the low solubility of PTX due to its bulky polycyclic structure hampers its clinical application [1]. Many attempts have been made to find less toxic and better-tolerated carriers to increase the solubility of PTX for intravenous delivery, such as nanoparticles, dendrimers, liposomes and nanosuspensions [2,3,4,5]. In recent years, polymeric micelles have attracted growing interest due to their attractive characteristics, such as their excellent solubilization ability, small size, high stability, prolonged circulation time, low toxicity, ability to evade scavenging by the mononuclear phagocyte system (MPS), high biocompatibility and efficient accumulation in tumor tissues via an enhanced permeability and retention (EPR) effect [6,7]. The micelles have a unique core–shell structure with hydrophobic segments as the internal core and hydrophilic segments as the outer shell. The internal core provides a storeroom for poorly water-soluble drugs, and the outer shell allows the retention of the stability of micelles in aqueous medium and provides the opportunity to target the delivery of antitumor drugs to the tumor by further modification [8,9].

To date, numerous amphiphilic block or graft copolymers have been synthesized and applied as micellar drug delivery systems [10]. Among them, chitosan (CS) has been extensively studied for its biocompatibility, non-toxicity and biodegradability [11,12]. Moreover, the abundant active amine and hydroxyl groups in CS could offer many opportunities for chemical modification. In recent years, several chitosan-based PTX delivery systems have been developed, such as *N*-mercapto acetyl-*N*′-octyl-*O*, *N*′′-glycol chitosan micelles [13], 3,6-*O*,*O*′-dimyristoyl chitosan micelles [14], folic acid–cholesterol–chitosan micelles [15], PTX conjugated trimethyl chitosan nanoparticles [16], palmitoyl chitosan nanoparticles [17], *N*-succinyl-chitosan nanoparticles [18] and PTX-loaded chitosan nanoparticles prepared by the nano-emulsion method [19]. However, chitosan with high molecular weight has poor solubility in aqueous medium at neutral pH, which limits its medical and pharmaceutical applications. In contrast, water-soluble chitosan with a low molecular weight and high degree of deacetylation is a superior candidate for amphiphilic copolymer synthesis [20]. In the present study, the water-soluble chitosan was used as the hydrophilic part of the copolymer to form a micellar system for PTX delivery.

Deoxycholic acid (DA) is a typical bile acid that is secreted from the gallbladder to emulsify fats and other hydrophobic compounds [21]. As an endogenous compound with a lipophilic nature, the introduction of DA to CS could adjust the hydrophilicity/hydrophobicity balance of the conjugate and would not lead to any serious toxicity [22]. DA has been approved as an excellent pharmaceutical additive for injection [23].

Molecular ligands were often grafted onto drug carriers to develop tumor-targeted drug delivery systems. It has been reported that folate receptors are over-expressed in many types of cancers, while almost undetectable in healthy tissues [24]. The folic acid-modified nanocarriers could improve therapeutic efficacy via folate receptor-mediated active targeting. The antitumor efficiency could be significantly enhanced by synergetic active and passive tumor targeting [25].

Based on the above, in present study, a biocompatible nanocarrier based on deoxycholic acid and folic acid-modified chitosan (FA-CS-DA) was designed for targeting the delivery of PTX. The synthesis, characterization and self-assembly of FA-CS-DA and the characterization and in vitro/in vivo antitumor activity of PTX-loaded micelles were studied in detail.

## 2. Results and Discussion

### 2.1. Preparation of FA-CS-DA

**Figure 1:**
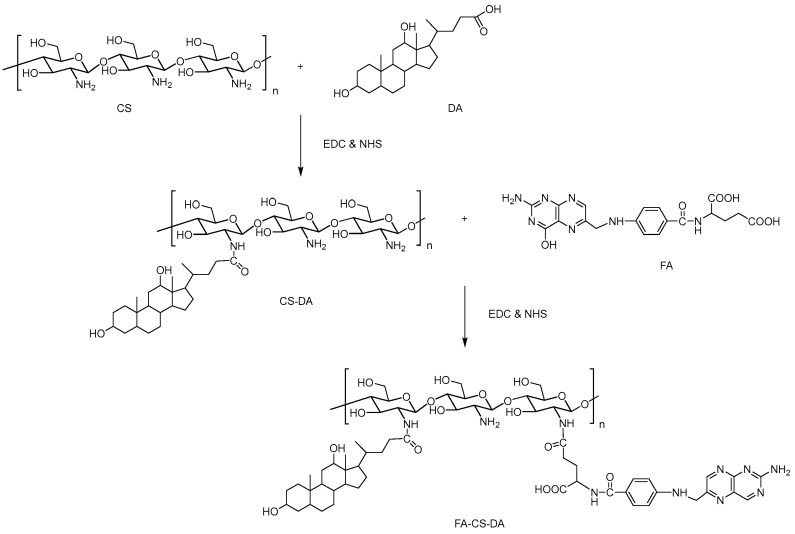
Scheme of the synthesis of FA-CS-DA (folic acid–chitosan–deoxycholic acid).

The synthesis of FA-CS-DA was performed via the amide bond formation between the amino groups of CS and the carboxyl groups of DA and FA. As shown in Figure 1, the FA-CS-DA was synthesized by a two-step reaction. First, the intermediate CS-DA was prepared by the conjugation of carboxylic groups of DA with the primary amino groups of CS. Then, an FA molecule was introduced by attaching the carboxyl groups of FA to the remaining terminal amino of CS-DA. EDC and NHS were used in both reactions to active the carboxyl groups [26]. The unreacted DA and FA, as well as any by-product, were removed by dialysis. The degree of substitutions (DS) of DA and FA were calculated as 15.8% and 8.0%, respectively.

### 2.2. Characterization of FA-CS-DA

#### 2.2.1. Proton Nuclear Magnetic Resonance (^1^H-NMR) Characterization

**Figure 2:**
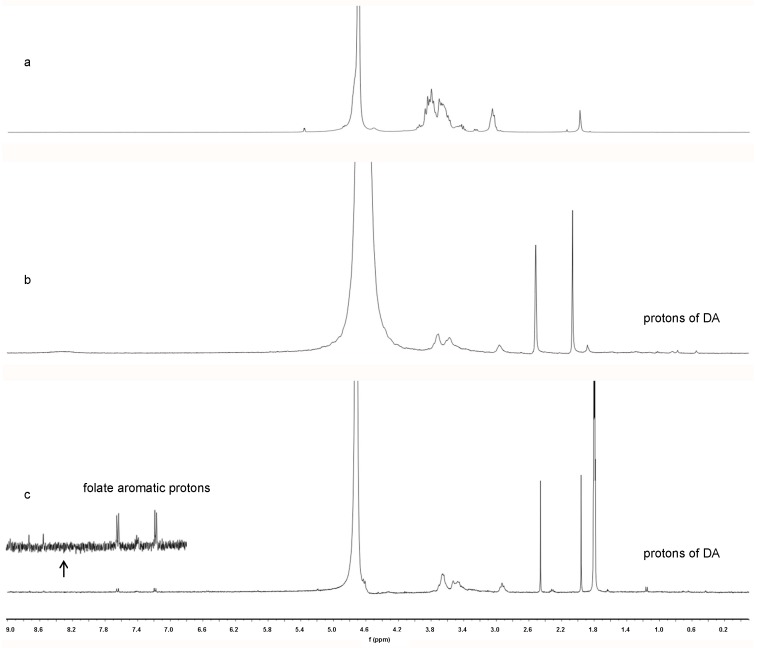
^1^H-NMR spectra of (**a**) CS, (**b**) CS-DA and (**c**) FA-CS-DA.

To confirm the conjugate formation, the ^1^H-NMR spectra of CS, CS-DA and FA-CS-DA are shown in Figure 2. Compared with CS, new peaks appeared in the range of 0.5–2.5 ppm in the spectrum of CS-DA and were assigned to the –CH_3_ and –CH_2_– protons of DA, which indicated the successful introduction of DA. Furthermore, FA-CS-DA showed characteristic signals attributed to the protons of FA at 7.20, 7.66 and 8.55 ppm. More specifically, the signal at 8.55 ppm was assigned to the proton of the pterin ring of FA, and signals at 7.66 and 7.20 ppm corresponded to the aromatic protons of FA. The aforementioned results revealed that both DA and FA were successfully grafted onto the backbone of CS.

#### 2.2.2. Fourier-Transform Infrared (FTIR) Characterization

**Figure 3:**
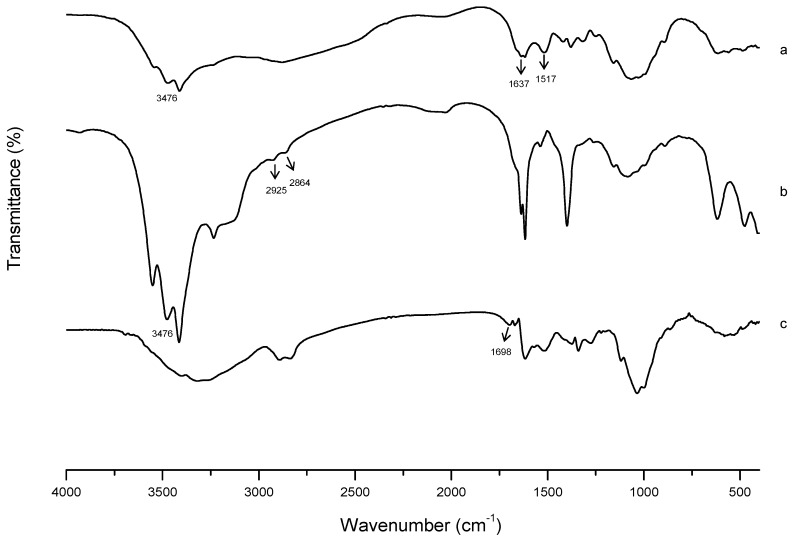
FTIR spectra of (**a**) CS, (**b**) CS-DA and (**c**) FA-CS-DA.

FTIR analysis was used to further confirm the successful synthesis of FA-CS-DA. As presented in Figure 3, for CS, the signal at 1637 cm^−1^ was attributed to the C–O stretching vibration of C=O group of the amide I band, and the peak at 1517 cm^−1^ was assigned to the N–H bending vibration of the amide II band. In the spectrum of CS-DA, new peaks at 2925 and 2864 cm^−1^ were due to the C–H stretching vibration of methylene of DA. The enhancement of peak intensity at 3476 cm^−1^ suggested the increase of hydroxyl groups after the grafting of DA. For FA-CS-DA, the new signal at 1698 cm^−1^ was assigned to the unreacted carboxyl groups in FA, which implied the introduction of FA. All these differences indicated the formation of FA-CS-DA.

#### 2.2.3. Critical Micelle Concentration (CMC) of FA-CS-DA

**Figure 4:**
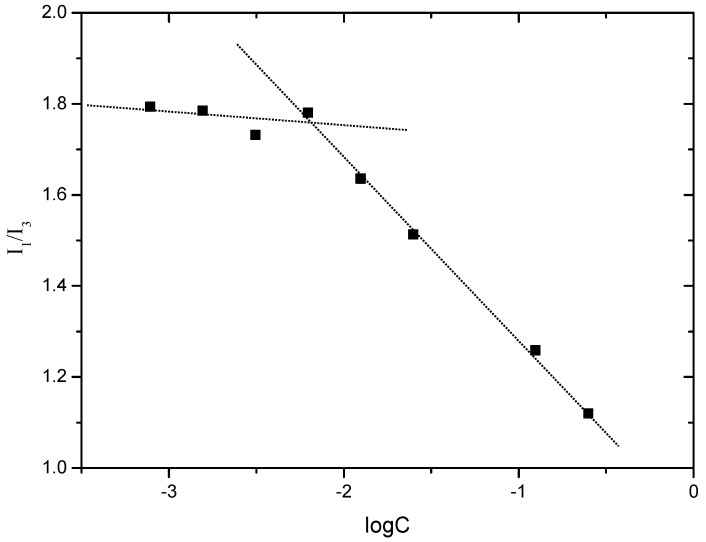
Variation of the fluorescence intensity ratio of I_1_/I_3_ against the logarithm of FA-CS-DA concentration.

CMC is the lowest concentration for the amphiphilic polymer to form micelles in aqueous medium, and it is an important parameter that indicates the stability of micelles. In this study, pyrene was used as a fluorescence probe to measure the CMC of FA-CS-DA. At low concentrations, the polymer molecules existed in a single-stranded form, and the fluorescence intensity remained constant. Once the concentration was higher than CMC, the polymer molecules formed micelles, and pyrene was solubilized in the hydrophobic core of micelles. As a result, the fluorescence intensity increased significantly. Moreover, the intensity of the third energy peak (383 nm, I_3_) increased more dramatically than the first peak (373 nm, I_1_) [27]. The intensity ratio of I_1_/I_3_ was used as an indicator of the polarity of the environment, and the variation of I_1_/I_3_ against the logarithm of polymer concentration is shown in Figure 4. The CMC of FA-CS-DA could be determined from the point of inflection, and the value was calculated to be 6.6 × 10^−3^ mg/mL. The low value suggested the high stability of FA-CS-DA micelles.

### 2.3. Preparation of PTX-Loaded FA-CS-DA Micelles

As the polymer FA-CS-DA had an amphiphilic structure, including hydrophobic segments of DA, hydrophilic segments of CS and tumor targeting ligands of FA, in an aqueous environment, it could self-assemble into micelles. In this study, FA-CS-DA was dispersed in distilled water, and the micelles were prepared under ultrasonication without the addition of any emulsifier or stabilizer. The drug loading into the hydrophobic domains occurred simultaneously with the formation of micelles via either hydrophobic–hydrophobic interactions or Van der Waals interactions between PTX molecules and the hydrophobic groups of the polymer [28]. For the optimized drug-loaded micelles, the drug encapsulation efficiency and the drug loading capacity were calculated to be 81.2% and 9.1%, respectively.

### 2.4. Characterization of PTX-Loaded FA-CS-DA Micelles

#### 2.4.1. Particle Size and Zeta Potential

It was well known that the particle size and size distribution of nanoparticles could dramatically affect the fate of the particles. Nanoparticles in the range of 10–200 nm could reduce the reticuloendothelial system (RES) uptake and enhance the endocytic uptake in tumors via the EPR effect [29]. The mean particle size of PTX-loaded FA-CS-DA micelles determined by the DLS method was 126 nm, with a polydispersity index (PDI) of 0.256, and this was larger than the bare ones (78 nm, PDI of 0.232), which indicated the encapsulation of PTX into the micelles.

Zeta potential is often used to indicate the stability of particle systems. With high zeta potential, the particles could repel each other and prevent aggregation, therefore enhancing the stability of the solution. The resultant zeta potential values of bare and PTX-loaded FA-CS-DA micelles were +29.1 mV and +19.3 mV, respectively. The relatively high positive potential was attributed to the ionized amino groups of CS. It was reported that the positively charged particles could enhance the endocytosis by cells [30].

#### 2.4.2. Transmission Electron Microscopy (TEM) Observation

**Figure 5:**
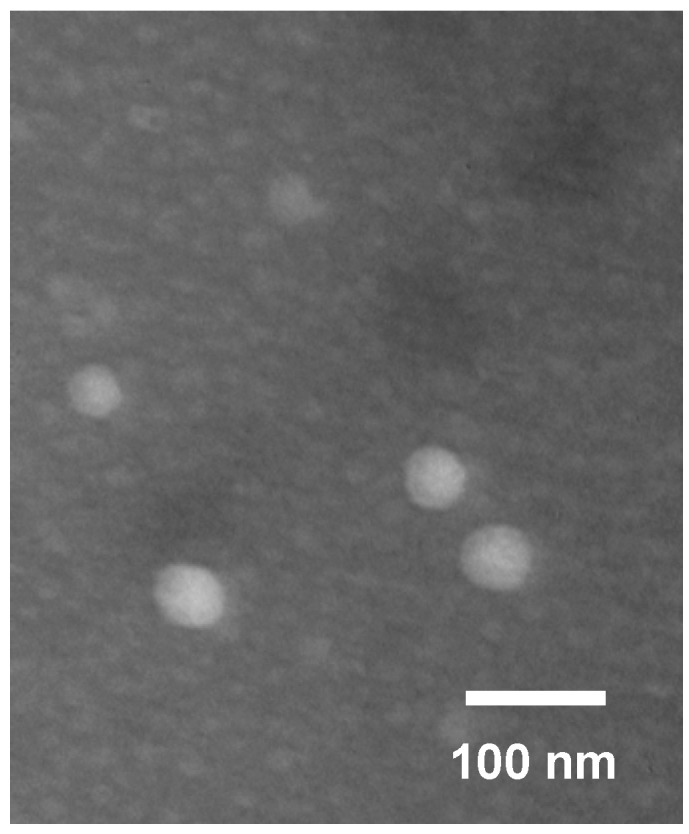
Transmission electron microscopy (TEM) image of paclitaxel (PTX)-loaded FA-CS-DA micelles.

TEM was used to directly visualize the size and morphology of the micelles. The TEM micrograph of the PTX-loaded micelles presented in Figure 5 showed that the FA-CS-DA was capable of forming polymeric micelles, and the micelles had a near-spherical shape with narrow distribution. Furthermore, the size obtained by TEM was smaller than that measured by DLS, which was due to the different states of the particles in the measurements, i.e., the dried state and the hydrated state, respectively. More exactly, the outer shell of the micelles could be collapsed during the process in TEM experiment [31].

#### 2.4.3. X-Ray Diffraction (XRD) Analysis

**Figure 6:**
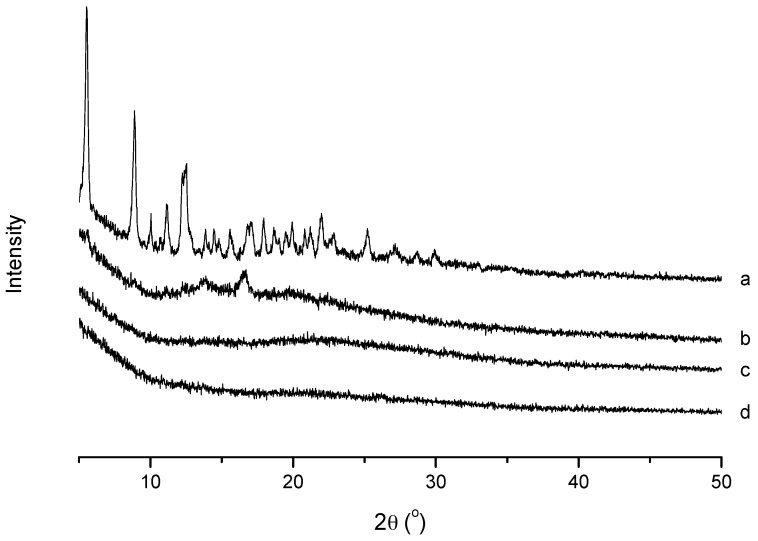
XRD spectra of (**a**) PTX, (**b**) a physical mixture of PTX and blank micelles, (**c**) PTX-loaded micelles and (**d**) blank micelles.

XRD analysis was conducted to confirm the existence state of PTX in the polymeric micelles. As shown in Figure 6, the XRD diagram of PTX presented several peaks at 2θ of 5.53°, 8.87°, 10.04°, 11.14° and 12.53°, and there were a large number of small peaks in the range of 15° to 30°. For blank micelles, there were no typical crystal peaks in the pattern. The physical mixture of PTX and bare micelles still showed the typical crystal peaks of PTX with weaker intensity. However, the PTX-loaded micelles had a similar spectrum to the blank micelles and there were no PTX peaks. It was implied that PTX was entrapped in the FA-CS-DA micelles in an amorphous or molecular state, which might lead to better absorption of the drug.

### 2.5. In Vitro Cytotoxicity Study

**Figure 7:**
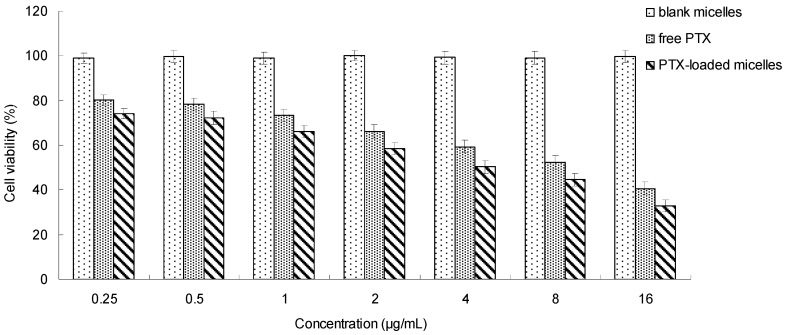
In vitro cytotoxicity of PTX-loaded FA-CS-DA micelles.

The in vitro cytotoxicity of the PTX-loaded micelles was evaluated by a standard MTT assay against MCF-7 cells. As illustrated in Figure 7, more than 99% of the cells were alive after the treatment of blank micelles even with high concentrations, which suggested the nontoxicity of the vehicle. For PTX formulations, the cytotoxicity was concentration-dependent. When the drug concentration increased, the cell viability decreased, which implied that a sufficient exposure level was important for the drug to kill the cells effectively. Moreover, it was exciting to see that the PTX-loaded micelles exhibited higher cytoxicity than the free PTX in dimethyl sulfoxide (DMSO). This might be explained by the effect of the FA-CS-DA micellar vehicle. For MCF-7 cells with over-expressed folate receptors on the surface [32], more FA-CS-DA micelles could be internalized into the cells via the receptor-mediated endocytosis. It could be speculated that FA-CS-DA might be a potential drug carrier for PTX.

### 2.6. In Vivo Tumor Growth Inhibition Study

**Figure 8:**
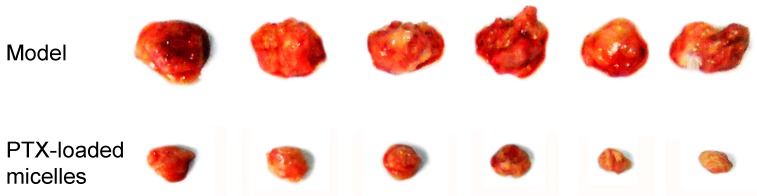
Tumors excised from the mice after intravenous injection treatment.

The tumor growth inhibition study was performed to further evaluate the in vivo antitumor activity of PTX-loaded FA-CS-DA micelles. As shown in Figure 8, the tumors excised from the mice in the PTX-loaded micelles group were significantly smaller than those from the normal saline group, and the TIR was as high as 78.1%. The outstanding antitumor efficacy could be explained by the following facts: first, there were a number of FA receptors on the surface of the tumor, and the PTX-loaded FA-CS-DA micelles could accumulate in the tumor tissues and then internalize into the tumor cells via the folate receptor-mediated active targeting. Second, via the electrostatic interaction, the positively charged micelles could efficiently bind to the tumor surface, which exhibited a high negative charge [33]. Moreover, the particles with positive charge were more likely to penetrate into the tumor cells [34]. Third, the particle size of <200 nm could facilitate the tumor accumulation of micelles by the EPR effect. In summary, all of these could increase the PTX concentration in the tumor, therefore obtaining excellent antitumor efficacy.

## 3. Materials and Methods

### 3.1. Materials

Chitosan (CS, Mw of 30 kDa, deacetylation degree > 90%) was obtained from Kittolife Co., Ltd., Seoul, Korea. Paclitaxel (PTX) was purchased from Natural Field Biological Technology Co., Ltd., Xi’an, China. Deoxycholic acid (DA), folic acid (FA), 1-Ethyl-3-(3-dimethylaminopropyl) carbodiimide hydrochloride (EDC) and *N*-hydroxysuccinimide (NHS) were supplied by Aladdin Industrial Co., Shanghai, China. Pyrene, 2,4,6-trinitrobenzene sulfonic acid (TNBS) and 3-(4,5-dimethylthiazol-2-yl)-2,5-diphenyl tetrazolium bromide (MTT) were supplied by Sigma Chemical Co., St. Louis, MO, USA. Dulbecco’s modified Eagle’s medium (DMEM), penicillin–streptomycin mixture and fetal bovine serum (FBS) were obtained from Gibco BRL, Carlsbad, CA, USA. All other chemicals and solvents were of analytical or chromatographic grade and used without further purification. Distilled water or Milli-Q water was used in all experiments.

### 3.2. Animals and Cell Lines

Specific pathogen-free mice weighing 20 ± 2 g were supplied by the Laboratory Animal Center of Harbin Medical University, Harbin, China. MCF-7 cells (human breast cancer cells) and H22 cells (mouse hepatocellular carcinoma cells) were kindly donated by the Department of Pharmacology, Harbin Medical University. All animal procedures were performed in compliance with the animal care protocols approved by the Animal Ethics Committee of Harbin Medical University (16 March 2018, No. 201803160028).

### 3.3. Synthesis of FA-CS-DA

FA-CS-DA was synthesized via the reaction of carboxyl groups of DA and FA with amino groups of chitosan under the catalyzation of EDC and NHS. The CS-DA was prepared as follows. Firstly, 1.0024 g of chitosan was dissolved in 50 mL of distilled water as solution A. The solution of DA (0.6012 g) with EDC (0.3426 g) and NHS (0.4113 g) in 50 mL of methanol was stirred for 1.5 h as solution B. Then solution B was added drop-wisely into solution A and stirred for 24 h at room temperature. After that, the reaction mixture was added to 50 mL of methanol. The resultant was filtered, and the filtrate was transferred into the mixture of methanol, water and ethanol at the ratio of 1:1:1 (*v*/*v*), then centrifuged at 6000 rpm for 20 min to remove unreacted DA and other impurities. The product of CS-DA was dried under vacuum at 40 °C.

For the synthesis of FA-CS-DA, a certain amount of CS-DA (0.14 g) was dissolved in 50 mL of acetate–acetate buffer solution (pH 4.7) as solution C. The solution of FA (0.04 g) with EDC (0.035 g) and NHS (0.042 g) in 20 mL of DMSO was stirred for 1 h as solution D. The solution D was added drop-wisely into solution C and stirred at an ambient temperature in dark condition. Twenty-four hours later, the resultant was dialyzed against an excess amount of distilled water (MWCO of 30 kDa) for 48 h for purification and then lyophilized to get the product of FA-CS-DA.

### 3.4. Characterization of FA-CS-DA

#### 3.4.1. ^1^H-NMR Characterization

^1^H-NMR spectra were recorded on a Bruker Avance NMR spectrometer (AV-400, Bruker, Switzerland) operated at 400 MHz to analyze the structure of FA-CS-DA. The mixture of deuterated water (D_2_O) and DMSO-d6 was used to prepared native and modified chitosan solution, respectively.

#### 3.4.2. FTIR Characterization

The formation of FA-CS-DA was further verified by FTIR spectra that recorded with KBr pellets using FTIR spectrometer (Tensor II, Bruker, Switzerland) in the range from 4000 to 400 cm^−1^, with a resolution of 2 cm^−1^.

#### 3.4.3. Measurement of the Degree of Substitution (DS)

The DS is defined as the number of DA (or FA) groups per 100 sugar units of CS. The DS of DA was determined by measuring the free amino groups of native chitosan and CS-DA via the TNBS method [35]. The DS of FA was measured by an ultraviolet-visible (UV-vis) spectrophotometer. Specifically, FA-CS-DA was dissolved in the mixture of water and DMSO at the volume ratio of 1:1, and the absorbance was determined by an UV-vis spectrophotometry (UV mini-1240, Shimadzu, Kyoto, Japan) at 363 nm. To get the standard curve for FA, a series of FA solutions with increasing amounts of FA were prepared.

#### 3.4.4. Determination of Critical Micelle Concentration (CMC)

The CMC of FA-CS-DA was determined by measuring the fluorescence intensity of pyrene using a fluorescence spectrometer (F-2500 FL Spectrophotometer, Hitachi Ltd., Tokyo, Japan). In brief, 10 μL of pyrene in acetone solution was added into a series of 10 mL volumetric flasks, and the acetone was evaporated in dark. Then, the FA-CS-DA solutions with different concentrations were added to each flask, and the final concentration of pyrene was 6 × 10^−7^ mol/L. The samples were sonicated for 1 h, and then remained undisturbed and kept from light to reach equilibrium. The emission spectra of pyrene were recorded from 350 to 500 nm at the excitation wavelength of 337 nm. The slit widths for excitation and emission were set at 5 and 2.5 nm, respectively. The fluorescence intensity ratio of the first peak (I_1_, 373 nm) to the third peak (I_3_, 384 nm) was analyzed for determination of CMC.

### 3.5. Preparation of PTX-Loaded FA-CS-DA Micelles

The PTX-loaded FA-CS-DA micelles were prepared by ultrasonication using a probe-type sonicator (JY92-II, Ningbo Scientz Biotechnology Co., Ltd., Ningbo, China) [20]. Firstly, FA-CS-DA was dispersed in distilled water. Then the PTX in acetone solution was added quickly, and the mixture was sonicated at 224 W for 10 min with 2 s active/3 s duration. The mixture was dialyzed against distilled water for 5 h by using a dialysis bag (MWCO of 30 kDa) to remove unloaded PTX. The resultant was subsequently freeze-dried (Freeze Dryer Model FD-1A-50, Bioyikang Experimental Instrument Co., Ltd., Beijing, China) to get the PTX-loaded FA-CS-DA micelles powder.

### 3.6. Characterization of PTX-Loaded FA-CS-DA Micelles

#### 3.6.1. Measurement of Particle Size and Zeta Potential

A dynamic light scattering method was applied to measure the particle size, zeta potential and size distribution of the samples using the Zetasizer^®^ 3000 (Malvern Instruments, Southborough, MA, USA).

#### 3.6.2. TEM Observation

TEM observation of the PTX-loaded micelles was performed using transmission electron microscopy (H-7650, Hitachi Ltd., Tokyo, Japan) operated at 60 kV. Before observation, an aqueous droplet of the sample was deposited on a copper grid coated with carbon. After 4 min, the grid was tapped with filter paper to remove surface water, followed by air drying and negatively stained with 2% phosphotangstic acid.

#### 3.6.3. XRD Analysis

XRD analysis was used to study the existence state of PTX after loaded into micelles. Samples were measured by an X-ray diffractometer (Geigerflex, Rigaku Co., Tokyo, Japan) using Cu Kα radiation source at 30 kV and 30 mA. The relative intensity was recorded in the range of 5–50° (2θ) at scanning speed of 4°/min and step size of 0.02°.

### 3.7. Determination of Drug Loading and Drug Encapsulation Efficiency

The amount of PTX encapsulated into the micelles was determined as follows: a certain amount of lyophilized drug-loaded micelles was weighted, and then acetonitrile was added, followed by ultrasonication at 200 W for 10 min to extract PTX from the core of the micelles. After filtered through a 0.22 μm microporous filter, the PTX concentration was measured by HPLC method using a mobile phase delivery pump (LC-10ATVP, Shimadzu, Japan) and a Diamonsil^TM^ C_18_ reverse-phase column (200 mm × 4.6 mm, 5 μm, Dikma Technologies Inc., Beijing, China). The mobile phase was the mixture of acetonitrile and water at a volume ratio of 70:30. The column temperature was set at 30 °C. The detection wavelength was 227 nm, and the flow rate was 1 mL/min. The drug loading (DL%) and drug encapsulation efficiency (EE%) were calculated with the following formulas.

DL% = W_encapsulated_/W_micelles_ × 100% (1)

EE% = W_encapsulated_/W_fed_ × 100% (2)

where W_encapsulated_, W_fed_ and W_micelles_ represented the weight of PTX encapsulated in the micelles, the PTX fed initially, and the weight of PTX-loaded micelles, respectively.

### 3.8. In Vitro Cytotoxicity Study

The cytotoxicity of PTX-loaded micelles was investigated by MTT method. H22 cells in the logarithmic growth phase were seeded in a 96-well microtitre plate at the density of 1 × 10^5^ cells/well and cultured in DMEM with 10% (*v*/*v*) fetal bovine serum and 1% penicillin–streptomycin at 37 °C in a humidified incubator with 5% CO_2_. When the cells became 75% confluent, free PTX, blank FA-CS-DA micelles and PTX-loaded FA-CS-DA micelles with a PTX concentration ranging from 0.25 to 16 µg/mL was added, respectively. After incubation for 24 h, the cell viability was determined. At a predetermined time, the supernatant of each well was aspirated off and replaced with fresh medium, and then 10 µL of MTT solution was added. With the action of active mitochondrial dehydrogenase in live cells, the dissolved MTT could be converted to water-insoluble purple formazan crystals. After incubation for another 4 h, the unreacted MTT was discarded, followed by the addition of 100 μL of DMSO to dissolve the formazan crystals. The absorbance was analyzed by a BioRad microplate reader (Bio-Rad 680, Bio-Rad Laboratories, Hercules, CA, USA) at 490 nm. Untreated control cells were set as 100% viable. Cell viability was calculated by the following equation:

Cell viability (%) = A_sample_/A_control_ × 100% (3)

where A_sample_ and A_control_ were the absorbance of cells exposed to the sample and the absorbance of untreated cells, respectively.

### 3.9. In Vivo Antitumor Activity Study

The in vivo antitumor activity of PTX-loaded FA-CS-DA micelles was evaluated in H22 tumor-bearing mice. To establish the tumor model, mice were inoculated subcutaneously in the right armpit with 5 × 10^6^ cells (0.2 mL) [36]. When the tumor xenografts became palpable, the mice were randomly divided into 2 groups (*n* = 6) and treated with physiological saline and PTX-loaded FA-CS-DA micelles (15 mg/kg), respectively. Samples were administered via the tail vein once every 3 days for 4 times. After 12 days of treatment, the mice were sacrificed and the tumors were harvested and weighted. The antitumor activity of the PTX-loaded micelles was expressed by the tumor inhibition rate (TIR), which could be calculated using Equation (4).

TIR = (1 − W_1_/W_2_) × 100% (4)

where W_1_ and W_2_ represented the average tumor weight of PTX-loaded micelles group and normal saline group, respectively.

### 3.10. Statistical Analysis

Each experiment was performed in triplicate. Values were expressed as mean ± standard deviation. Statistical data analysis was performed using the Student’s *t*-test with the significance level set at *p* < 0.05.

## 4. Conclusions

In the present study, an amphiphilic chitosan derivative of FA-CS-DA was synthesized via amidation reaction. The polymer could self-assemble into micelles in an aqueous milieu and showed good solubilization ability for PTX. The developed micellar system had excellent features, such as high stability upon dilution, high drug loading and encapsulation efficiency, small particle size and excellent cytotoxicity to tumor cells in vitro and in vivo. It could be concluded that the FA-CS-DA was a promising micellar carrier for PTX delivery, and further, more detailed studies will be performed.
